# Management perspectives from patients with fibromyalgia experiences with the healthcare pathway: a qualitative study

**DOI:** 10.3389/fmed.2023.1231951

**Published:** 2023-12-01

**Authors:** Alexandra Kachaner, Magda Harim, Alice Combier, Anne Priscille Trouvin, Jérôme Avouac, Brigitte Ranque, Marie-Aude Piot

**Affiliations:** ^1^Service de médecine interne, Université Paris Cité, Paris, France; ^2^Service de réanimation, Hôpital Henri-Mondor, Créteil, France; ^3^Université Paris Cité, Service de Rhumatologie, Hôpital Cochin, Paris, France; ^4^Université Paris Cité, Centre d'Évaluation et de Traitement de la Douleur, Hôpital Cochin, Paris, France; ^5^Université Paris Cité, Service de Médecine Interne, Hôpital Européen Georges Pompidou, Paris, France; ^6^Université Paris Cité, Hôpital Necker Enfants Maladies, AP-HP, Paris, France

**Keywords:** fibromyalgia, qualitative research, general practice, somatic symptom disorder, chronic pain

## Abstract

**Background:**

Fibromyalgia is a prevalent condition affecting 1–2% of the general population and can result in significant disability. Physicians and patients frequently encounter challenges in managing this condition.

**Aim:**

The aim of this study was to explore novel management approaches through a qualitative analysis of the doctor-patient relationship.

**Design and setting:**

Telephonic interviews were conducted with fibromyalgia patients to investigate their healthcare experiences.

**Methods:**

Qualitative analysis was performed on patients' narratives using interpretative phenomenological analysis, a methodology that delves into each individual's subjectivity.

**Results:**

A total of 19 adult patients with fibromyalgia, primarily middle-aged women (84% women, mean age 49.8 years), recruited from two university centers in Paris, were included in the study. The narratives of participants revealed substantial suffering and considerable functional impairment, which is paradoxical for a condition often considered benign. They reported an ongoing sense of loss of control, exacerbated by an imbalanced patient-doctor relationship. Patients constantly feared not being heard or believed, and they frequently sought attention from their caregivers. Most participants displayed significant ambivalence toward the nature of their condition and actively sought causal links. Patients' adaptive strategies sometimes worsened their symptoms, as in the case of muscular deconditioning. The healthcare system appeared deficient in managing these patients, characterized by a lack of health professional training, frequent inappropriate responses from healthcare providers, and stigmatization of psychological conditions.

**Conclusion:**

Despite its perceived benign nature, fibromyalgia should be regarded as a severe condition due to its substantial long-term consequences. Participants reported a challenging experience with the doctor-patient relationship, marked by a strong sense of dependence and a lack of recognition. The care pathway for these patients appeared unsuitable and disorderly, potentially resulting in iatrogenic consequences. The management of patients with fibromyalgia should be enhanced and directed toward a patient-centered approach. The study provides practical recommendations regarding communication methods and patient care.

## Introduction

Somatic symptom disorder (SSD) is a very common condition, affecting 6% of the general population and 17 to 30% of patients consult a general practitioner (1, 2). The nosological framework is complex, with multiple overlapping denominations (somatoform disorders, medically unexplained symptoms, bodily distress syndrome), and it comprises several subtypes such as fibromyalgia, irritable bowel disease, or chronic fatigue syndrome, illustrating the difficulty doctors have in understanding this pathology (3). SSD is defined in the DSM-V by one or more symptoms having a significant functional impact, behaviors, thoughts, or feelings that appear disproportionate to the symptoms, and chronic evolution (4). The treatment-resistant symptoms, the frequent complex personality and/or psychiatric co-morbidities, and the absence of an identified anatomical substratum negatively impact the doctor-patient relationship. Most doctors feel that patients with SSD are hard to manage (5).

Among these disorders, fibromyalgia is an entity characterized by diffuse musculotendinous pain and excessive emotional burden. Old definitions associated the presence of widespread pain with the finding on physical examination of tenderness in at least 11 of the 18 points of palpation (6). It affects 1–2% of the general population, with a strong female predominance and varying degrees of severity up to complete disability (7, 8). Its pathophysiology is poorly understood. Psychosocial factors are involved in a frequent context of psychological distress. For example, an association between the incidence of fibromyalgia and job-related stress and dissatisfaction has been shown (9). It seems also strongly linked with past psychological trauma (10–12). There is evidence for alteration of the pain control centers, both in the peripheral and central nervous systems, and for a genetic component in the pathogenesis of fibromyalgia (13–15). Fibromyalgia can be triggered by infections (16, 17). A poor current understanding of the pathogenesis makes management guidelines difficult to establish. Treatment options rely on antidepressant drugs (18), and there is moderate evidence for mind- and body-based therapy and physical exercise (19, 20). Some patients with fibromyalgia are waging a real battle for legitimacy with political and socio-economic concerns (reimbursement of care, insurance coverage, over-medicalization, etc.), which calls into question the place of this disease in their lives and in society in general (21).

This condition often leads to severe disability, deterioration of quality of life, and work stoppage, and results in high healthcare resource utilization and medical nomadism (4). Moreover, due to diagnostic wandering, it has a tremendous cost for society, sometimes greater than that of serious illnesses with well-defined management (10). To date, in France, dedicated management cannot be provided for all patients suffering from this pathology.

As caregivers, we felt the need to improve the management of this disease. Several studies have reported qualitative data on the experiences of persons with fibromyalgia, with a global approach, exploring different aspects of this condition (22–24), but to the best of our knowledge, no study has explored in depth the complexity of their interactions with the healthcare system. In a qualitative study, 76% of interviewed general practitioners found fibromyalgia time-consuming and frustrating (25). The patient-doctor relationship can then become a detrimental circle (26). The aim of this study is to perform a qualitative analysis of their doctor-patient relationship, through a first-person approach to look for new management methods for fibromyalgia tailored to the patient's experience.

## Materials and methods

This qualitative study conforms with the COREQ 32 criteria (27).

### Theoretical framework

Qualitative analyses were based on the IPA (interpretive phenomenological analyses) method, which aims to understand how people make sense of their major life experiences by adopting an “insider perspective” (28). IPA is underpinned by three epistemological points: first, phenomenology explores the participants' views of the world (29); second, hermeneutics deals with interpretation in a double manner: “researcher is trying to make sense of the participant trying to make sense of their world” (30, 31); third, idiography emphasizes a detailed and in-depth understanding of each individual case. This method is particularly suited to studies that focus on the subjectivity of individuals and is often chosen for the study of complex phenomena, such as chronic pain. Further details on researcher reflexivity are reported in Supplementary material 1. Data triangulation was performed, and recruitment stopped when data sufficiency was obtained (Supplementary material 1).

### Participants

We conducted a reasonably purposive homogeneous sampling of adult persons who had experienced fibromyalgia. The study took place from June 2021 to December 2021 in two centers of the university hospital group APHP Paris Center: the internal medicine department of Georges Pompidou Hospital and the pain management department of Cochin Hospital. Participants with a diagnosis of fibromyalgia established by their referring physician were consecutively included during an outpatient consultation with their referring physician. They were given an information note about the aim and modalities of the study. Oral consent was obtained. They received no remuneration for their participation. The sample size rationale is detailed in Supplementary material 2.

### Data collection

Participants were contacted according to their availability and interviewed following a guide (Supplementary material 3). Interviews were recorded, and the main elements were transcribed verbatim.

Following IPA recommendations, we stopped the patient's recruitment when all researchers agreed that the data were sufficient to ensure an adequate and in-depth understanding of the patient's experience (32).

### Analyses

The verbatim transcripts were analyzed inductively, moving progressively from the descriptive to the interpretative level with consideration of the empirical data.

First, the interviews were completely replayed, read, and re-read to get an overall impression. Initial notes were taken with the participants' own words, including several themes, discourse modalities, and the researchers' first interpretations about the participants' intentionality. Special attention was paid to language elements such as rhythm, metaphors, and expressions because these helped to understand the phenomenological level, that is, authentical intentionality.

To ensure validity, separate analyses were conducted and then compared by two researchers. Then, in the second stage, themes were determined inductively by searching for recurrences of meanings that made sense to participants while addressing the research questions. Two researchers (an internist and expert in fibromyalgia and a psychiatrist trained in qualitative research) triangulated the initial analyses by helping to compare and synthesize emergent and cluster themes.

Then, themes were gathered in a third stage named the “matricial stage,” which creates super-ordinate themes addressing the research questions directly for each participant.

Each record was analyzed separately in the first instance following this in-depth process. They were then compared with one another, creating the fourth level and ultimately the results.

Interpretative features were active at each level of analysis. However, the main researchers tried to limit their effect, as reported in the section on reflexivity (Supplementary material 1). The work was reviewed by three rheumatologists. Every discrepancy was negotiated during study group meetings, and the final organization emerged from this collaborative work.

### Ethical agreement

The study was approved by a French IRB (Avis 2019-A030116-51 du Comité de Protection des Personnes Sud Est VI).

## Results

All patients with fibromyalgia who were offered to participate in the study agreed to do so. A total of 19 participants were included: 84% (16/19) were women, and their mean age was 49.8 years (SD ± 14.6 years). All participants met the DSM-5 SSD criteria and the ACR diagnostic criteria for fibromyalgia (33). They were all French-white patients. The common psychological characteristics of the participants are detailed in Supplementary material 4. Interviews lasted a median of 46 min (IQR 43–65 min). Results were organized into three super-ordinate themes: the first dealt with perceived vulnerability to an unrecognized disease (I), the second explored answers concerning a healthcare system perceived as rejecting (II), and the third described responses for dealing with this condition (III). Themes and citations are summarized in Supplementary material 5.

### Perceived vulnerability to an unrecognized disease

#### The paradox between an apparently benign illness and a major alteration in quality of life

Participants described unbearable symptoms using torture terminology, for example Participant 15 (female, 60–65 years) said “I was on an electric chair”. The perceived handicap appeared worthy of advanced chronic illnesses, as expressed by Participant 2 (female, 40–45 years): “I have half a life, half a day” or by Participant 3 (female, 50–55 years): “I feel like a bedridden patient”. A large proportion of participants had experienced work loss, pay cuts, and a reduced social circle. This seemed so important that some participants even expressed the “hope” of having another diagnosis, a more palpable illness that would be acknowledged by medicine and provide the opportunity for treatment, even if it was associated with organ damage. At the same time, they mentioned their good health condition as Participant 3 (female, 50–55 years): “I was happy to know that I had no risk of ending up disabled”.

#### Intolerable experience of loss of control

Most participants described a high level of previous functioning and perceived control, lost with fibromyalgia, as reported by Participant 17 (female, 50–55 years): “I was very, very dynamic, I had incredible energy” or by Participant 3 (female, 50–55 years): “I had a lot of energy, I used it up and I have nothing left”. Some participants had fluctuating and unpredictable symptoms that prevented them from planning anything, which led to social and professional withdrawal. In that context, the asymmetric doctor-patient relationship was often mentioned by patients as increasing their loss of control through a feeling of dependence. Consequently, many participants reported a loss of self-esteem and confidence. This condition made the patients feel that they had shifted from being actors to observers.

#### Difficulties accepting the diagnosis

The diagnostic announcement was a key moment that seemed to influence the subsequent clinical course. Most of the participants interviewed seemed to have difficulties accepting it or even just making it their own, as mentioned by Participant 2 (female, 45–50 years): “Making this diagnosis does not help to solve it” or by Participant 18 (female, 40–45 years): “It's unbearable socially speaking, at least people who break their leg can say so”. Most participants also showed ambivalence about accepting it or exploring other avenues, fearing that a differential diagnosis could have been missed. Some patients even felt ashamed of this diagnosis, particularly when a relative had a “more serious” illness, which prevented them from talking about it. This complicated communication and altered their relatives' support. Conversely, a few patients reported good clinical improvement due to the diagnostic announcement, but they were a minority.

#### Ambivalence toward psychological issues

Almost all the participants expressed a reluctance to receive psychological care. They considered the psychological difficulties related to this diagnosis stigmatizing, as if it questioned the reality of their symptoms, as Participant 12 (female, 25–30 years) admitted: “I was told what I did not want to hear”. During narratives, this reluctance manifested as a fluent emphasis on somatic symptoms, while discourse was disrupted when addressing psychological difficulties. Some of them avoided questions about psychological difficulties, interrupting the dialogue when these questions were raised, refusing psychiatric referral, while others agreed to see psychiatrists or psychologists but denied psychological issues, as evoked by Participant 1 (male, 35–40 years): “Yes, I had a psychological shock, actually, I don't feel like that but my wife and my mother…”.

### Answers concerning a rejecting healthcare system

#### High expectations and disappointment with the healthcare provider

Participants described physicians in terms of emotional breakdown and abandonment, attentional and emotional needs, and a fear of being rejected, as Participant 3 (female, 50–55 years) said: “I kept saying ‘What a pity he's gone'.”

Participants described good attention from the doctor in the initial phase, when the symptoms were attributable to a potentially serious somatic cause, but the quality of listening was perceived as having deteriorated once the doctor understood they were dealing with fibromyalgia. Participants sometimes reported tactless attitudes, e.g., if the doctor made the patient feel that he was wasting his time, as Participant 8 (female, 25–30 years) reported: “He told me that I had to stop worrying about life and that because I was a woman, I had a mental burden”.

All participants voiced a fear of not being heard and believed by doctors, feeling rejected by them, as mentioned by Participant 9 (female, 40–45 years): “I was the hypochondriac” or by Participant 7 (female, 60–65 years): “You don't have anything, it's nothing, it's in your head”. They expressed a need for reassurance and reported experiencing regression and dependence on doctors. During the research interview, almost all had pressured speech. We interpreted it as a concern for exhaustiveness, making the medical researcher feel that they sought at all costs to be believed, despite the lack of somatic evidence. They used suspenseful effects, trying to convince or impress, e.g., by using very strong images or self-mockery, to make themselves more credible.

Participants reported that this conflictual doctor-patient relationship had encouraged them to seek out new practitioners with medical nomadism. Sometimes the doctor's lack of empathy was pointed out as incompetence, indicating that the patient was looking for emotional support.

#### Knowledge gap of healthcare workers delaying appropriate care

Participants described several consultations where fibromyalgia was misunderstood and its management poorly codified. The mean diagnostic delay exceeded ten years in our sample.

Participants expected doctors to acknowledge their doubts instead of maintaining a dogmatic position in this situation of uncertainty, as Participant 12 (female, 25–30 years) expressed: “The GP was honest enough to tell me that he did not understand, and I found that very professional of him”. They criticized the restrictive setting of the consultation, not adjusted to their complex problems, as explained by Participant 9 (female, 40–45 years): “we are only allowed to come for one ailment, not more” or by Participant 11 (female, 80–85 years): “You are young, miss, but you know, doctors used to know how to take their time with patients”.

The other healthcare workers were also pointed out for their incompetence. Some participants mentioned psychologists who were unfamiliar with this condition, as Participant 12 (female, 25–30 years) pointed: “the psychologist I have, we have a good relationship, but at times I feel she is a bit lost”. Others reported attitudes with iatrogenic consequences, such as a physiotherapist telling a patient that she had probably had a stroke, which could increase anxiety. In addition, patients reported frequent situations of accidental mistreatment by healthcare workers, which are not specific to this condition but can contribute to the patient's negative experience.

#### Perceived deficits in the organization of the healthcare system

The feeling of not being heard encouraged participants to make multiple attempts to meet “the” doctor who would be able to help them, as Participant 14 (female, 35–40 years) said: “I was looking on the internet for specialists who are experts in my symptoms”. The lack of health professional training on this condition was also highlighted by many participants. All these shortcomings seemed to be iatrogenic for them, as their anxiety and malaise increased with the absence of appropriate follow-up.

### Responses for dealing with this condition

#### Reactions appearing to increase perceived suffering

Some responses seemed counterproductive because they ultimately exacerbated symptoms. For example, Participant 7 (female, 60–65 years) limited her physical activity to prevent tiredness, yet this seemed to increase global daily fatigue, with possible muscular deconditioning. She explained: “Exercising makes me very tired. I don't want to be even more tired, so I stay on the couch.” Some participants also developed anticipatory anxiety, i.e., watching for the onset of their symptoms, and excessive focus on the slightest sensation. They carried out an excessive number of complementary examinations, and some of them recognized that it increased symptom-related anxiety. Some participants expressed a feeling of fatality and pessimism, limiting coping resources, as reported by Participant 4 (female, 50–55 years): “When I plan something, I call to cancel because I know I will be tired, so I avoid it”.

#### Combative attitude to regain control

Some strategies aimed to regain control of the situation. In the diagnostic process, many patients conducted their own investigation, practicing significant medical nomadism, as Participant 8 (female, 25–30 years) argued: “When you're not well, you look for something, it's a question of survival”. They could refute one doctor's opinion with another's. They sometimes positioned themselves as doctors, appropriating the medical discourse with technical terms and medical explanations, as Participant 12 (female, 25–30 years) said: “I had the dose lowered” [of medication]. Some participants were strongly argumentative, using complex reasoning to illustrate their points or even showing aggressiveness to avoid feeling dominated. Several participants also reported a combative attitude with a strong desire to return to their previous state. Thus, some of these strategies for regaining control could have deleterious effects and make the doctor-patient relationship complex because the patient's defensive reaction may be difficult to understand.

#### Strategies that seemed to alleviate psychological distress

Being supported appeared to have a major impact, whether by family, friends, or other patients, as expressed by Participant 5 (female, 65–70 years): “I know where to find help”. Other participants found resources in certain traditional or alternative therapies, such as adapted physical activity, as Participant 18 (female, 40–45 years) explained: “I was advised to do Chi Gong. It's great, I'd recommend it to anyone, anyone who has a stressful job. Then I did Pilates and stretching, and I felt a bit better”. Some participants managed to combine somatic and psychological approaches; Participant 4 (female, 50–55 years) even said that psychological treatment would have saved her some imaging tests: “To do less scanning, I have to do more relaxation”. Despite their reluctance, most participants reported some satisfaction with an intervention at some point by a psychologist or a psychiatrist, as Participant 14 (female, 35–40 years) expressed: “She referred me to CBT and it was such a relief”. Finally, several participants described strategies for the acceptance and alleviation of symptoms with “reasonable” goals, which seemed to allow for gradual improvement.

## Discussion

### Summary

We chose the IPA method to explore the relationship between patients with fibromyalgia and the healthcare system because it allows for a deep exploration of the subjectivity of each experience. Analysis of the participants' narratives showed that they experienced major disability, with a feeling of rejection and dependence on the healthcare system. They tried to implement their own solutions with varying degrees of success.

### Comparison with existing literature

This study emphasizes the extent of these patients' suffering and disability caused by an illness often considered benign (34). This paradox is reflected in the DSM-5 definition of somatic symptom disorder, which defines this condition through its very significant impact (4). In our study, patients almost constantly feel unrecognized and often rejected by doctors. Indeed, doctors frequently lack empathy toward patients with SSD (35), which questions their illness representation. The current biomedical model focuses mainly on biological deficits to assess the severity of an illness, while the person-centered approach stresses the importance of functional impact (36). Therefore, a person-centered approach probably makes more sense in managing fibromyalgia and SSD in general.

We assume that the study interview reproduces the framework of the usual medical consultation, as researchers are doctors. Patients often seemed anxious, emotionally overwhelmed, and had pressured speech. Participants' pressured speech has three possible and not mutually exclusive interpretations: 1) the patient may not want to give voice to the doctor for fear that he will put an end to the interview; 2) it may be a manifestation of anxiety; or 3) a concern for completeness in participants who often seem to have perfectionist traits. For doctors, dealing with these patients is complex, with significant negative countertransference experienced by the researchers during these interviews. They may find it difficult to have an empathetic attitude toward a pressured speech, sometimes emotionally overcharged patient who requires a long consultation. One study showed that anxious physicians who work more than 55 h/week were more likely to find emotionally overwhelmed patients difficult to manage, indicating that sometimes mental availability is lacking in these overworked and stressed physicians (37).

In our study, participants were very reluctant to verbalize their emotions, even though their speech was prolix and filled with somatic problem descriptions. This may be a consequence of alexithymia, as this trait is more common in patients with fibromyalgia and SSD in general (38, 39). The origin of the problems always seems to be external to the patient. This contrasts with the fact that the participants' narratives reveal numerous traumatic clues and even some possible post-traumatic stress disorders. These elements are frequent among patients with SSD (2–4). An amalgam is sometimes made by both doctors and patients between what is psychological and what does not exist, which complicates communication, as illustrated by this obviously traumatic expression cited by almost all participants: “it is all in your head.” One may ask whether the physical symptom becomes a vehicle for the expression of psychological suffering because of the denial of psychic issues. In a world in which psychiatric problems are denigrated and stigmatized in comparison with somatic diseases, the body can become the means of expression for psychological suffering.

### Limitations

Patients are being enrolled in a university hospital environment, which may have favored the recruitment of severe forms and failures in outpatient care. The doctor-patient relationship previously built between the participants and the physicians who recruited them could have influenced their consent to participate. Despite careful precautions to make the participants feel at ease, the researchers' medical status could also have limited the free speech of our participants and the triangulation process, even if we mixed specialties.

Of note, the small size of our sample is not a limitation, as IPA does not seek representativeness or data saturation but rather a homogenization of the sample because it is a phenomenological approach that is embodied and contextualized (Supplementary material 3).

### Implication for practice

The analysis of narratives from persons with fibromyalgia emphasized their suffering, their disability, and the perceived lack of recognition from the medical profession, highlighting the need to improve their care and to change the approach of caregivers. Based on issues emerging from the patients' narratives, we provided practical guidelines to help physicians improve their practice (Table 1), suggesting management strategies and examples of communication modalities that rely on a patient-centered approach. Given the high prevalence of SSD and the iatrogenic effects of poor medical management, we believe that all general practitioners should receive specific training to help them understand and manage the condition properly.

**Table 1:** Practical recommendations arising from the study.

**Themes**	**Suggested attitude**
**Perceived vulnerability to an unrecognized disease**	
The paradox between an apparently benign illness and a major alteration in the quality of life	Make this paradox explicit. Recognize explicitly the suffering of the patient. Point especially the difficulty of not being taken seriously by doctors and the family or friendly environment.
Intolerable experience of loss of control	Empower the patient through positive reinforcement and avoidance of dogmatic attitudes. Look for the positive effect of some voluntary behaviors (e.g. disappearance of symptoms during physical exercise) or life experiences (inducing mental distraction).
Difficulties in accepting the diagnosis	Name the condition. Point the high prevalence of such disorders and make the difference with psychiatric conditions. Provide pathophysiological explanations about the unbalanced involuntary functioning of the central nervous system rather than point to an external cause of distress. Have the patient rephrase the diagnostic, and unmask resistances to adopt it.
**Answers of healthcare system perceived as rejecting**	
Difficulties in building the therapeutic alliance with disappointed high expectations with the medical figure	Work on modalities of communication, and mobilize empathy toward this disabling condition. Spend more time on the first consultation, or reschedule a long consultation, before moving to shorter ones.
Fear of not being heard and believed, with anxious pressured speech	Make the patient write down its symptoms before the consultation and ensure him/her this list will be inserted in his/her medical file. However, discourage permanent monitoring of the symptoms, which enhances attentional focus on them.
Knowledge gap of health workers delaying appropriate care	Acknowledge one's own uncertainty toward the causes of the condition but propose plausible pathophysiological explanations, such as cerebral distress with unbalance toward sensitive pathways and involuntary hypervigilance to bodily signals and hyperactivation of autonomous nervous system.
**Development of responses for dealing with this condition**	
Excessive medical nomadism	Reassure the patient on the absence of organ damage that could entirely explain the symptoms. Point the futility and the stressful role of further explorations and multiple referrals even if an unknown or rare underlying condition can never be totally ruled out. Ensure him/her that the medical work-up will be resumed if the condition worsens.
Muscular deconditioning	Supervised adapted physical activity, with very progressive intensity and duration. Reassure the patient about transitory episodes of fatigue after physical activity and respect thresholds to avoid subsequent flairs of the symptoms. Help him/her choose a physical activity or sport that he/her enjoys.
Absence of pharmacological treatment	Develop patient coping strategies, especially relaxation methods and positive reinforcement to abandon deleterious avoidance strategies (physical activity or social isolation). Do not discourage reasonable self-treatment but warn against constant monitoring of the symptoms and excessive medical research, especially on the Internet. Expose the proven psychological and physical benefits of physical activity. Propose cognitive behavioral therapy if necessary.
Need for psychological support	Screen for anxiety and depressive symptoms. Don't directly ask about past traumas but suggest it may be one of the causes once a good patient/doctor relationship is established. Refer to a psychiatrist if necessary while maintaining a somatic follow-up (with a referral letter mentioning the diagnosis of somatic symptom disorder).
Patient social isolation	Refer to a social worker, and encourage the patient to seek support from family members or patients' associations. Do not encourage prolonged work stopping but rather suggest part-time work if possible.

## Data availability statement

The raw data supporting the conclusions of this article will be made available by the authors, without undue reservation.

## Author contributions

AK, MH, BR, and M-AP contributed to the conception and design of the study. AK and MH interviewed the patients and analyzed them under the supervision of M-AP and BR. AK wrote the first draft of the manuscript. JA, AT, and AC revised the manuscript. All authors fulfill the criteria for authorship, contributed to the manuscript revision, read, and approved the submitted version.
