# Supplementary material for: Management perspectives from patients with fibromyalgia experiences with the healthcare pathway: a qualitative study
Source: Front Med (Lausanne). 2023 Dec 1;10:1231951. doi: 10.3389/fmed.2023.1231951 (PMC10722233; doi:10.3389/fmed.2023.1231951)
Supplement: Supplementary file 1 [file Data_Sheet_1.DOCX]

**Appendixes**

**Supplement 1: Reflexivity**

To improve data triangulation, the qualitative analysis was performed by a team of four physicians: MAP (psychiatrist), MH (general practitioner), BR and AK (internists) – intentionally multidisciplinary to improve the triangulation process. AC, JA, and APT (rheumatologists) reviewed the manuscript and provided an outside perspective on the analytical work.

Regarding the four principal investigators:

They are four women, physicians, and work in different Parisian academic hospitals.

Interviews were conducted by MH and AK, who are medical residents.

MH and AK are residents in general medicine and internal medicine, respectively; they had little experience with this pathology at the beginning of the study. To BR and MAP, this seemed to be a favorable condition to approach this phenomenon, as it was being studied with a naïve view. In addition, they both kept a method journal to spell out preconceived ideas before the interviews began; they also achieved this through challenging discussions during research meetings. They were trained in qualitative methodology by MAP.

The research project was the subject of their medical theses.

BR is a professor of medicine in a university internal medicine department. She has extensive clinical and research experience in this illness. She instigated this work because, seeing many patients in consultation, she had sensed the need to improve the understanding of the experience of this illness. Thus, she assumed that her involvement toward improving this illness may influence this study.

Some participants of our study were also patients of BR. However, careful attention was paid to ensure strict anonymization so that the participants included in the study could not be recognized by BR, thus preventing influence on care.

MAP is a pediatric and adolescent psychiatrist in a university pediatric psychiatry department. She is trained in qualitative methodology and has already conducted, supervised, and published qualitative work, including several works using the IPA method (1–3). She had no contact with the participants before and during the study. She is used to working with adolescent patients expressing their psychic suffering through their body in a psychiatric unit focusing on eating disorders. Her psychiatric specialty provided a complementary view of the participants’ narratives compared with other researchers more familiar with somatic diseases.

MAP trains residents in qualitative research through seminars and exercises included in basic training. In this context, she trained MH and AK while providing further articles and feedback to conduct the whole research process. IPA was the first method with which she was trained herself. As reported in her first publication including IPA (3), she was trained in the Epidemiological and Public Health Research Center, INSERMU1178/CESP U1018, Villejuif (France) by Drs. Jean-Sebastien Cadwallader, Massimiliano Orri, Juliette Gueguen, and Prof. Bruno Falissard, who themselves have conducted and published qualitative works using IPA.

The four researchers share a common inclination for a comprehensive and patient-centered approach, which led to the realization of this work. They believe in the importance of the patient’s subjective experience in healthcare and in the importance of co-constructing therapeutic solutions, especially in situations where evidence-based medicine lacks clear answers. Therefore, the diagnostic criteria chosen for the study focus on the impact of the symptoms and their experience, and they embody a holistic approach. Indeed, the definition of somatic symptom disorder (SSD) in the DSM-5 follows this path, contrary to numerous symptom-listing scales that can be found in functional disorders. The whole research process is therefore informed by the four researchers’ patient-centered approach.

The researchers were aware of the potential complications between the setting of the traditional medical consultation and the research situation.

Moreover, it was realized that the two main researchers’ medical status in itself could influence the patients’ confidence in developing their narrative: either with positive (as they could do with their practitioners) or negative effects (inhibitions, reminders of previous bad doctor-patient relationship experiences). Thus, a particular effort was made so that the patient would feel at ease from the beginning of the study. A short introduction clarified the aim while setting a benevolent framework for their participations. Interviews were constructed in a way that allowed the patients to express themselves freely, with very open questions at the beginning. The questionnaire was written in a manner that influenced the patient as little as possible. Care was taken to use empathetic language to facilitate communication.

The patients were not involved in the analysis of the data, which would seem to be a sensitive issue outside a psychotherapeutic setting.

**Supplement 3: Data collection**

Participants were contacted according to their availability. A short introduction reminded them of the study’s aims while trying to make them feel as comfortable as possible. Semi-structured interviews were conducted flexibly following a guide (Supplement 4) based on the researchers’ experience and literature research, including both expertise on fibromyalgia (BR) and on mental health and qualitative research (MAP).

According to IPA, the concept of “data saturation” and preconceived sample size does not make sense. Indeed, the notion of saturation – as described by Morse (4) – implies both no new information emergence from data and a broad and diversified sampling while being driven by categories and concepts emerging from iterative analysis (5).

In IPA, the patient sample must be homogeneous regarding the experience of participants with the phenomena studied. The small size of our sample is not a limitation, as IPA does not seek representativeness or data saturation, but rather a homogenization of the sample, because it is a phenomenological approach that is embodied and contextualized.

However, recruitment should ensure an adequate and in-depth understanding of patients’ experiences as recommended (6). It was stopped when all researchers agreed that they had reached sufficiency. That is, the collection was stopped when the researchers felt that they had gathered sufficient information and had an adequate in-depth understanding of the phenomena regarding narrowness of research aim, belonging to a specific target group, quality of dialogue, and depth of analysis (case by case) (7).

This assumes a subjective researcher judgment while ensuring that they had conducted a homogeneous sample regarding the phenomena of interest. To limit subjectivity that could influence this judgment, the researchers tried to improve triangulation by including several medical specialties, diversifying the views which made decisions.

**Supplement 3: Interview guide**

“Good morning/evening, madam/sir. My name is Magda/Alexandra. I am calling about the study that your referring physician told you about and for which we made an appointment. The purpose of this study is to trace the care pathway of patients with diffuse pains. I would like to give you the floor so that you can tell me about your journey and then I will ask you some more targeted questions. There are no right or wrong answers. If there is something you don’t understand, don’t hesitate to tell me, I will rephrase it. Our interview is recorded, and the data you provide will remain completely anonymous. Do you have any questions before we begin the interview?”

1. Can you tell me about the different steps that led to your current diagnosis?

Potential follow-up:

- How was your relationship with the doctors you met?

- Since the first consultation, how has your relationship with the doctor who is currently treating you evolved?

(Evaluate, without naming them, the confidence in the different doctors, the conviction of being a victim of medical errors, the hurtful or benevolent behavior of the doctor, the doctor’s listening skills)

- When did the symptoms begin and what symptoms did you have? What were the most disabling symptoms?

- When did you first seek care for these symptoms?

2. Can you tell me about the fibromyalgia diagnosis?

Potential follow-up:

- How did you feel when you were told about this?

- What did you think about this diagnosis?

- Have your thoughts about the diagnosis changed?

- When was the diagnosis of fibromyalgia first mentioned and affirmed? Who made the diagnosis? How convinced were you of the diagnosis of fibromyalgia?

- When did you continue your follow-up with the same medical team?

3. What is the impact of this disease on your life?

Potential follow-up:

- In your family? In your professional environment? In the broader social field?

4. What medical history did you have at the time of diagnosis?

5. Have you experienced any significant physical trauma in your lifetime? (car accident, orthopedic trauma with severe pain as a result, etc.)

If yes, what type?

6. I will now ask you a rather personal question, which you are free not to answer. To what extent do you think you have been a victim of psychological trauma and/or lack of affection in your life?

Potential follow-up:

Have you been a victim of physical, verbal, or sexual abuse?

To what extent would you say you have experienced abandonment in your life?

7. Do you have any comments to add?

8. What would you suggest to improve the diagnosis of fibromyalgia?

**Supplement 4: Common features associated with the experience of fibromyalgia**

A- Common psychological background

The participants interviewed showed similarities in their presentations and attitudes. A particular psychological profile emerged with recurring traits, without asserting a causal or consequential link. Among these recurring features was very frequent anxiety, sometimes with panic attack–like descriptions, and anticipatory anxiety. Many participants also seemed to have a very perfectionist profile, with a concern for detail and exhaustivity in their speech, a behavior that sometimes evoked the “model student,” and a high level of previous functioning and requirements. Some participants appeared to have depressive symptoms such as severe apragmatism, overt sadness with crying during the interview, or the expression of overwhelming despair. Lastly, we suspected that some participants had addictive behaviors, particularly regarding painkillers and possible eating disorders (weight variation, multiple food avoidances, etc.).

B- Frequent reporting of physical or psychological trauma

Participants frequently reported past physical or psychological trauma, ranging from physical aggression to profound emotional deprivation, sometimes evolving since childhood. Some participants evoked post-traumatic elements such as avoidance or flashbacks. Several participants presented neurological symptoms (inability to move a limb, neuropathy-like pain) shortly after the diagnosis of a neurological disease in a relative, raising the possibility of “mimicry,” in a situation where the illness of the loved one may have been an emotional shock. Finally, a certain number of traumas were directly related to care, either physical traumas linked to diagnostic or therapeutic procedures or repeated micro-traumas during medical consultations subsequently causing anxiety. In addition, many participants experienced abandonment, feeling both socially isolated and neglected by healthcare professionals.

C- Major importance of the social and professional environment

The social isolation and the absence of a supportive environment seemed to be correlated with the severity of the disease, with a causal link that could be interpreted in either direction. Concerning the professional environment, the participants often evoked possible work-related triggers to their symptoms. They were often off work or on disability because of their symptoms, even though they described their jobs as being central in their lives. This might have led to self-depreciation and loss of identity, which could have contributed to the negative impact of this condition.

| **Superordinate themes** | **Themes** | **Example** | **Citation** |
| --- | --- | --- | --- |
| Perceived vulnerability to an unrecognized disease | Paradox between an apparently benign illness and a major alteration in quality of life | Lexicon of torture | "Electric chair"  "Fifty thousand different pains" |
|  |  | High perceived handicap | "I have half a life, half a day"  "I feel like a bedridden patient" |
|  |  | Conscience of their good health condition | "I was happy to know that I had no risk of ending up disabled" |
|  |  | « Hope » of differential diagnosis | "It wasn't hope, but I was waiting for someone to give me the little blue pill, that they were going to find it and tell me how to move on, and I didn't want to be told that it was psychological" |
|  | Intolerable experience of loss of control | High level of previous functioning | "I was very, very dynamic, I had an incredible energy" |
|  |  | Loss of self-esteem and confidence | "I had a lot of energy, I used it up and I have nothing left" |
|  | Difficulties in accepting the diagnosis | Lack of treatment | "Making this diagnosis does not help to solve it" |
|  |  | Shame of the diagnosis | "It's unbearable socially speaking, at least people who break their leg can say so" |
|  | Ambivalence toward psychological issues | Rejection of psychological involvement | "I was told what I did not want to hear" |
|  |  | Marked difficulty to express emotions | "Yes, I had a psychological shock, I don't feel like that but my wife and my mother…" |

**Supplement 5: Synthesis of ideas into superordinate themes and themes**

**Supplement 5 : Synthesis of ideas into subtopics and topics**

| **Superordinate themes** | **Themes** | **Sub-themes** | **Citation** |
| --- | --- | --- | --- |
| Answers concerning a healthcare system perceived as rejecting | High expectations and disappointment with the healthcare provider | Attentional and emotional quest towards doctors | "I kept saying "What a pity he's gone"  " I was given the opportunity to see the professor in private "  " a young psychiatrist, a very handsome man " |
|  |  | Doctors negative countertransference | "He told me that I had to stop worrying about life and that because I was a woman I had a mental burden" |
|  |  | Constant pressured speech | "Do you have 3-4 hours for the list? "  "I always find it hard to talk about my pains because it's endless, I try to write, but I'm a bit ashamed with my endless lists of stuff" |
|  |  | Narrative strategies to convince | "Others would have committed suicide"  "I woke up feeling like I had spent six months in a cemetery". |
|  |  | Poor patient doctor relationship encourages medical nomadism | "I didn't find a doctor like the one I had before... I really looked but I didn't find the relationship I had before" |
|  | Knowledge gap of health workers delaying from appropriate care pathway | Doctors are confronted to an uncontrolled situation and cannot keep their traditional attitude of conviction | "The GP was honest enough to tell me that he did not understand, and I found that very professional of him"  " I feel more confident with people who are searching than with people who are too categorical" |
|  |  | The timing of traditional consultation is too short | "we are only allowed to come for one ailment, not more"  "you are young, Miss, but you know that before, doctors knew how to take their time with patients" |
|  |  | Issues are similar with other carers | "the psychologist I have, we have a good relationship but at times I feel she is a bit lost" |
|  |  | Medical nomadism is frequent | "I was looking on the internet for specialists who are experts in my symptoms" |
|  |  | Health carers have a lack of training in this condition | "The psychologist did not know how to conduct CBT for this indication"  "The doctor did not know about this illness" |

| **Superordinate themes** | **Themes** | **Sub-themes** | **Citation** |
| --- | --- | --- | --- |
| Development of responses for dealing with this condition | Reactions appearing to increase perceived subjective suffering | Muscular deconditioning with increased fatigue | " Exercising makes me very tired. So I don't want to be tired, so I stay on the couch." |
|  |  | Anxious hyperfocusing and anticipatory anxiety | "When I plan something I call to cancel because I know I will be tired, so I avoid it" |
|  |  | Coping skills are limited by a sense of inevitability | "I tell myself that I have to bear the pain [...]There will be nothing more in my life and that's it" |
|  | Combative attitude to regain control | Patients conduct their own investigation | "When you're not well, you look for something, it's a question of survival" |
|  |  | Defensive position of knowledge | "I had lowered the dose [of medication]." |
|  | Strategies that seemed to alleviate psychological distress | Being supported is comforting | "I know where to find help" |
|  |  | Adapted physical activity is a resource | "I was advised to do Chi Gong. It's great, I'd recommend it to anyone, anyone who has a stressful job. Then I did pilates and stretching and I felt a bit better" |
|  |  | Psychologic support seems helpful | "To do less scanning I had to do more relaxation"  "She referred me to CBT* and it was such a relief" |

*CBT: Cognitive Behaviour Therapy

1. Piot MA, Gueguen J, Michelet D, Orri M, Köenig M, Corcos M, et al. Personal recovery of young adults with severe anorexia nervosa during adolescence: a case series. Eat Weight Disord. 2020 Aug;25(4):867–78.

2. Kalindjian N, Hourantier C, Ludot M, De La Londe JG, Corcos M, Cadwallader JS, et al. Experiences of French medical students during their clerkship in adolescent psychiatry: a qualitative study. Eur Child Adolesc Psychiatry [Internet]. 2022 Feb 16 [cited 2022 Aug 17]; Available from: https://link.springer.com/10.1007/s00787-021-01940-1

3. Gueguen J, Piot MA, Orri M, Gutierre A, Le Moan J, Berthoz S, et al. Group Qigong for Adolescent Inpatients with Anorexia Nervosa: Incentives and Barriers. van Wouwe JP, editor. PLoS ONE. 2017 Feb 2;12(2):e0170885.

4. Morse JM. The Significance of Saturation. Qual Health Res. 1995 May;5(2):147–9.

5. Matthew B. Miles, A. Michael Huberman, Johnny Saldana,. Qualitative Data Analysis A Methods Sourcebook. fourth edition. 2019.

6. Smith J OM. Interpretative phenomenological analysis. Qualitative Psychology: A Practical Guide to Methods. London: SAGE; 2003.

7. Malterud K, Siersma VD, Guassora AD. Sample Size in Qualitative Interview Studies: Guided by Information Power. Qual Health Res. 2016 Nov;26(13):1753–60.
